# Current use and barriers and facilitators for implementation of standardised measures in physical therapy in the Netherlands

**DOI:** 10.1186/1471-2474-12-106

**Published:** 2011-05-22

**Authors:** Raymond AHM Swinkels, Roland PS van Peppen, Harriet Wittink, Jan WH Custers, Anna JHM Beurskens

**Affiliations:** 1 Zuyd University of Applied Sciences, Dept of Physical Therapy, Heerlen, The Netherlands; 2 Zuyd University of Applied Sciences, Centre of Research Autonomy and Participation for People with Chronic Illnesses, Heerlen, The Netherlands; 3 Utrecht University of Applied Sciences, Dept of Physical Therapy, Utrecht, The Netherlands; 4 Utrecht University of Applied Sciences, Research Centre for Innovations in Health Care, Utrecht, The Netherlands; 5 University of Applied Sciences, Amsterdam School of Health Professions ASHP Amsterdam, The Netherlands; 6 Dept Science, Royal Dutch Society for Physical Therapy (KNGF), Amersfoort, The Netherlands

## Abstract

**Background:**

In many countries, the need for physical therapists to use standardised measures has been recognised and is recommended in clinical practice guidelines. Research has shown a lack of clinimetric knowledge and clinical application of measurement instruments in daily practice may hamper implementation of these guidelines.

**Objectives:**

The aims of our study were a) to investigate the current use of measurement instruments by Dutch physical therapists; b) to investigate the facilitators and barriers in using measurement instruments.

**Methods:**

To get a complete and valid overview of relevant barriers and facilitators, different methods of data collection were used. We conducted a literature search, semi-structured interviews with 20 physical therapists and an online survey.

**Results:**

Facilitators are the fact that most therapists indicated a positive attitude and were convinced of the advantages of the use of measurement instruments. The most important barriers to the use of measurement instruments included physical therapists' competence and problems in changing behaviour, practice organisation (no room; no time) and the unavailability and feasibility of measurement instruments. Furthermore, physical therapists indicated the need to have a core set of measurement instruments with a short user's instruction on application, scoring and interpretation.

**Conclusions:**

The main barriers are on the level of the physical therapist (lack of knowledge; not focusing on the use of outcome measures) and organisation (lack of time; availability; lack of management support).

There seems to be a disparity between what physical therapists say and what they do. The majority of participating physical therapists indicated a positive attitude and were convinced of the advantages of the use of measurement instruments. However, the main problem for physical therapists is when to use which instrument for what patient (lack of knowledge). Furthermore, physical therapists indicated a need to compile a core set of measurement instruments with instructions concerning application, scoring and interpretation. Based on the identified factors, a number of strategies will be developed and evaluated in future studies.

## Background

**Figure 1:**
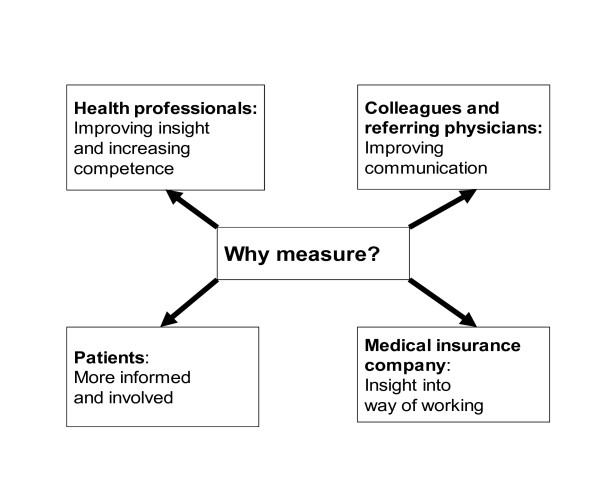
**Factors of relevance to use measurement instruments in clinical practice**.

In almost every disease, condition or ailment that receives attention in modern medicine, methods have been developed for describing or rating the observed clinical phenomena. Clinimetrics is the practice of assessing or describing symptoms, signs and laboratory findings by means of scales, indices, and other quantitative instruments [1]. Clinimetrics contributes to the process of clinical reasoning, objectifying and quantifying, which is essential for good clinical practice [2-4]. Physical therapists, therefore, use questionnaires, (pain)provocation-, performance - and observation tests for diagnostic, prognostic and evaluative purposes. The use of measurement instruments in clinical practice is relevant from four points of view (Figure 1), namely those of health professionals/doctors, colleagues, patients and medical insurance companies.

The need for physical therapists to use standardised (outcome) measures has been recognised worldwide and has been articulated in a number of policy statements, including the Core Standards of Physiotherapy Practice of the World Confederation for Physical Therapy (http://www.wcpt.org/node/29447). Not only do we need to evaluate physiotherapy treatment outcomes as an integral part of professional accountability, we need to make sure our diagnostic process is transparent, and that we are able to give our patients some sense of their prognosis with treatment. As a result, clinical practice guidelines often incorporate specific recommendations for the use of standardised measurements and measurement tools.

Over the last decade, the development of evidence-based clinical guidelines has been critical for quality improvement for the Royal Dutch Society for Physical Therapy (KNGF). This has resulted in the development and publication of 18 (mono-disciplinary) Clinical Practice Guidelines (CPGs). Many Dutch CPGs are translated into English. They can be downloaded at https://www.kngfrichtlijnen.nl/654/KNGF-Guidelines-in-English.htm.

As well as in guidelines developed in other countries, in these guidelines, the use of standardised measurement instruments is recommended in order to support the process of clinical reasoning. However, considerable variation in guideline adherence exists among therapists [5,6]. Research in the Netherlands has revealed that a lack of knowledge and competencies of physical therapists with respect to the use of measurement instruments may hamper the implementation of these clinical practice guidelines [7-11]. The data support the notion that these problems are present in more countries [12,13] and that there is room for improvement in the use of published measurement instruments in clinical practice [14-17].

Much research has been done on implementation methods of clinical practice guidelines in health care [5], but until recently [14,15,18] there were virtually no study findings on the implementation of measurement instruments in physical therapy clinical practice [11,12]. These more recent studies demonstrate a knowledge gap concerning measurement tools, resulting in the need for different methods to improve implementation of measurement tools. In the Netherlands, the implementation of the guidelines and measurement instruments was performed in a relatively passive way until 2008. The Royal Dutch Society for Physical Therapy made active implementation of measurement tools and practice clinical guidelines a key aspect of its quality policy (2007) and in 2008 started a project '*Measurement in clinical practice' *in cooperation with two research centres in Utrecht and Heerlen. The final goal of the project was to improve the use of standardised measures in physical therapy daily practice.

The project group adopted the model of systematic implementation by Grol & Wensing [19], comprised of the steps presented in Figure 1. Grol et al. emphasise that a thorough analysis of improvement goals and the current situation in the intended setting is essential for successful implementation. Strategies should be targeted at specific barriers and facilitators of the desired change.

### Step 1: Detection of improvement goals

Grol et al emphasise that the innovations to be implemented must be of good quality, fit in with the needs of the target group, be useable end easily available and be attractively designed [19].

### Step 2: Analysis of the target group and setting

This analysis concerns the characteristics of the target group, the factors that stimulate and hamper change and the aspects of performance that show the greatest deviation from the proposed behaviour. Factors that determine whether the implementation is successful or not may be connected to the setting in which the change is to be implemented, the relationship between individuals within the setting, the goals of the implementation, the professionals and the involved patients and the organisational or structural conditions.

### Step 3: Selection of the implementation strategies

The literature gives a great number of different strategies that can be used for the implementation of changes. Usually, a mix of strategies is selected, but the problem is that the most optimal choice is not only dependent on the strategy but also on the target group and its context.

### Step 4: Testing and execution of the implementation plan

When making an implementation plan, attention has to be paid to effective dissemination, both to encourage its acceptance and to promote the actual implementation and integration in normal working routines.

### Step 5: Evaluation and readjustment of the implementation plan

This step is crucial to be sure of the effectiveness of implementation. To find out whether the goals have been reached they must be made measurable. That means that outcome measures must be defined clearly to put into operation.

**Figure 2:**
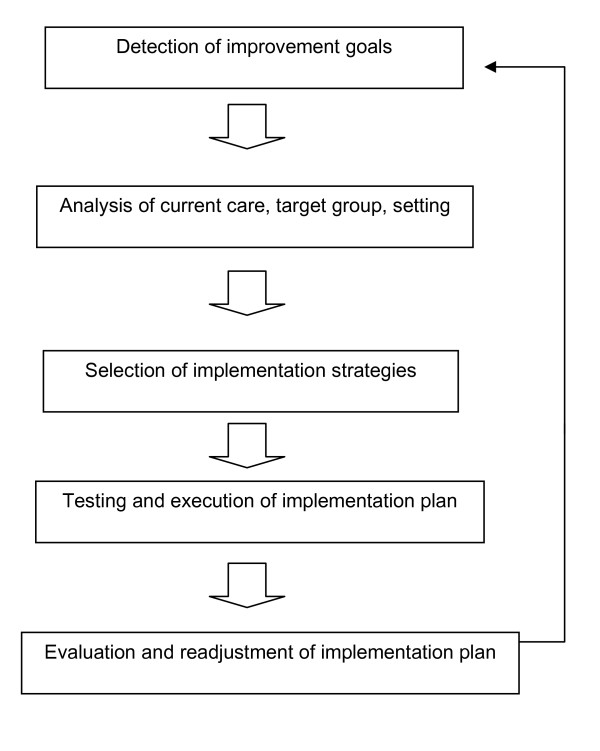
**Implementation model (Grol et al, 2005)**.

The project group followed steps 1 and 2 of the implementation model: a) investigating the current use of measurement instruments and b) investigating the facilitators and barriers in the use of measurement instruments. The project focused in particular on physical therapists working in two different settings: private practices and nursing homes. The reason to focus on the former group is that the majority of physical therapists in the Netherlands work in private practices. Physical therapists working in nursing homes were chosen because of the expected contrast in the kind of measurement tools used by private practice physical therapists. We also expected that the two different settings would require different implementation strategies. In this paper we describe the first two steps of the implementation model (Figure 2). The main goal is to examine the most important facilitators and barriers to the implementation of routine use of outcome measures. In continuation of this work, steps 3-5 will be evaluated after drawing up an inventory of the barriers and facilitators.

## Methods

To obtain insight into current measurement use and relevant facilitators and barriers, three methods of data collection were used. First, a literature search was performed for facilitators and barriers to implementation in health care in general and facilitators and barriers to implementation of measurement instruments in physical therapy practice in particular. Second, semi-structured interviews were used to identify facilitators and barriers to the use of standardised measures and how often which instruments are used. Third, we quantified these factors in an online survey of physical therapists.

### Data collection

#### 1. Literature search

A literature search was performed in the databases PubMed, CINAHL and Cochrane from inception until December 2010, on barriers and facilitators in health care in general, and implementation of measurement instruments in physical therapy practice in particular. The keywords used for the literature search are presented in Appendix 1. The reference lists of the identified articles were subsequently searched for additional studies. Out of results of the literature study a topic list was formulated for the interviews and online survey.

#### 2. Semi-structured interviews

Twenty semi-structured interviews were planed: ten interviews with physical therapists in the private sector and ten interviews with physical therapists working in nursing homes. Based on earlier interviews in a comparable study [11], it was assumed that more than twenty interviews would result in repetition of the same arguments (saturation) and lack of additional information. In the case of no saturation of answers, the number of interviewed therapists should be enlarged. Interviewed therapists were recruited by written letter from two different regions in The Netherlands, with 50% from each region. In the case of agreement to participate, an appointment was made for the interview. Selection of the therapists to be interviewed was based on the expected variation (theoretical sampling). For the selection, a distinction was made between physical therapists using measurement instruments regularly and therapists who did not use measurement instruments. Physical therapists were randomly selected from the KNGF member list of the two concerned regions. The interviewers were experienced qualitative researchers and physical therapists, external to the project group, with experience in the field of application of measurement instruments. Each interview took approximately one hour. The in-depth semi-structured interviews were digitally audio taped, summarised to the essentials and subsequently member checked. Interviewed colleagues authorised the text of the interviews. All participating therapists were KNGF members, which guarantees that they satisfied the quality requirements of the KNGF. 'Semi-structured' means that every interviewed physical therapist was asked the same questions, in an attempt to make the results optimally comparable. The following topics were discussed in the interviews:

- Knowledge of participants about the background (properties of instruments/tests) and current use of measurement instruments (familiarity with instruments in practice).

- Attitude and individual readiness about the usefulness of measurement instruments in practice and their actual use.

- Participants' opinions on the most important barriers and facilitators for implementation in daily practice as well as participants' thoughts on how best to overcome barriers for the implementation of a standardised set of instruments.

- The conditions needed for successful implementation.

In the interviews we also focused on underlying thoughts and possible solutions. The facilitators and barriers mentioned in the interviews were classified into four categories: 1) Physical therapist (competence and attitude), for example lack of knowledge; 2) Organisation (practice and colleagues), for example lack of time; 3) Patients, for example patients unaccustomed to the use of questionnaires and 4) Measurement instruments, for example overly extensive instruments. This classification is based on findings from another study where barriers to the use of standardised outcome measures were investigated [11]. The rationale for this classification was to be able to create optimal tools to improve the use of measurement instruments (steps 3-5 of the Grol model) on different levels, as mentioned in the categories above.

#### 3. Online survey

To compose the questionnaire for the survey we added relevant topics from the semi-structured interviews to the Barriers and Facilitators Questionnaire [7] (BFQ) (see Appendix 2). The survey (see Appendix 3) was (by random sampling) sent by email to 2900 physical therapists from two regions in the Netherlands selected from the member list (N = 16000) of the Royal Dutch Society for Physical Therapy (KNGF) (therefore, the sample included 18% of all members). The aim of this survey was to list how frequently which measurement instruments are used by physical therapists, which barriers and facilitators are reported most frequently and which facilitators might help with implementation.

##### Role of the funding source

This study was funded by the Dutch Scientific College of Physiotherapy (WCF) of the Royal Dutch Society for Physical Therapy (KNGF), with the final goal to perform a national structural implementation of the use of standardised measures in practice.

## Results

### Literature search

Many publications exist on implementation in general or implementation of guidelines in health care. Specifically, Cochrane et al. [20] identified seven categories of barriers: supports/resources (e.g., time, funding, resources), cognitive/behavioural (e.g., knowledge, awareness, skills), healthcare professional (e.g. characteristics, age/maturity of practice, peer influence), system/process (e.g., workload, team structure, referral process), attitudinal/rational-emotive (e.g., perceived competence, perceived outcome expectancy, authority), clinical practice guidelines/evidence for physical therapy (e.g., utility, access, local applicability), and patient factors (e.g., patient characteristics, adherence). A number of studies examined perceptions of the facilitators and barriers to using standardised outcome measures among rehabilitation professionals, and many of the reported barriers were similar across studies [3,4,7,10,13]. Lack of time for identification of a suitable measure, its administration and scoring and interpretation of results, lack of administrative support and resources, lack of financial compensation, lack of knowledge (familiarity with, lack of training in), lack of agreement on which measures to use and lack of access to measures were the most important barriers. Physical therapists' attitudes towards the use of standardised measurement instruments were found to be both a barrier (problems changing behaviour, fear of "cook-book practice" and a facilitator (a positive attitude towards standardized measures).

These findings mirror the seven categories of barriers as defined by Cochrane et al. [20] except for the patient factors. Perceived facilitators were knowledge of clinimetrics and support of colleagues in the use of measurement instruments [7], active educational initiatives, expertise and professional support, mandatory reporting of outcome measures [21] (at organisational and health care system levels) and having a Master's degree.

On the implementation of standardised measures, fewer studies were available [5,11,21,22]. Abrams et al. reported on a significant increase of standardised measure use by Australian physiotherapy providers to a transport accident scheme over a course of 6 months. In 2003, the Australian Physiotherapy Association (APA) adopted a national position statement on treatment justification that restated the professional requirement to measure outcomes using valid and reliable instruments and the Transport Accident Commission (TAC) produced a ''Clinical Justification Model'', a decision algorithm that included a requirement to use standardised measures to assess activities/participation and monitor outcomes for individual patients. The implementation of the clinical justification model was supported by a series of lectures and education seminars offered by the APA and TAC, educational material (including copies of a range of standardised questionnaires) was made available in hard copy and on the TAC and APA websites and peer contact was made with physiotherapy treatment providers to assist them implement the clinical justification model. An increase from 30-66% use of standardised activity and participation measures was found in a predominantly orthopaedic caseload [21]. Little change (41% to 43%) in the proportion of Canadian physiotherapists using ''published measurement scales'' was reported by Kay et al. [17] after physiotherapists were surveyed in 1992 and 1998, despite the publication of a battery of rehabilitation instruments and introductory outcome measures workshops. An increase from 30-50% to 100% in using standardised measures for mobility and balance by physiotherapists working with older people in Ireland was reported by Stokes and O'Neill [16]. Changes in the health care system, demanding more accountability and quality of service, were thought to contribute to this increase.

### Interviews

The semi-structured interviews were conducted with ten private practice physical therapists and ten nursing home physical therapists. No new information was gleaned in the last two interviews, indicating that the sample size was sufficient to saturate the topics.

There appeared to be minor differences between physical therapists working in nursing homes versus private practice (no financial compensation for use of outcome measures for physical therapists working privately; lack of support from management for physical therapists working in nursing homes), but the great majority of factors were comparable. These are summarised in Table 1. In both groups, physical therapists indicated lack of knowledge and understanding. In addition, therapists in both groups mentioned lack of time and unavailability of measurement instruments as important barriers.

**Table 1:** Barriers for the use of measurement instruments for the different categories, based on the semi-structured interviews (n = 20)

Level	Barriers
**Physical therapist**	

- competence	Lack of knowledge, education, routine and experience

	Focus of diagnosis on impairment

- attitude	Resistance to change

	Not being convinced of the added value of measurement instruments

	Being overloaded with information

	Headstrong in terms of own working method

	Defining the outcome of therapy in other ways

	Lack of confidence in own skills

**Organisation**	

- practice	Too much time investment

	Lack of financial compensation

	Lack of computers and digital questionnaires

	Absence of practice policy

- colleagues	Lack of discussions, meetings and feedback from colleagues

	No compliance with agreements made

**Patient**	

	Different expectations and preferences: patient are not familiar with measurement instruments and only want to be treated

	Patients cannot be tested because of problems with language, lack of cognition etc.

**Measurement instruments**	

	Poor availability of instruments

	Difficult to choose because of the large number of instruments

	Feasibility: extensive, difficult, interpretation, unclear instructions etc.

Most therapists indicated that the application of measurement instruments in daily practice is difficult and not yet integrated in the process of clinical reasoning.

During the interviews, the therapists were honest and admitted that they did not use the instruments as often as they would like to do. In particular, the semi-structured interviews indicated that application of measurement instruments is certainly not part of daily clinical practice.

In the interviews, physical therapists indicated needing a core set of measurement instruments with a short user's instruction on application, scoring and interpretation, as well as a need for small-scale tailor-made education and frequent feedback in order to facilitate their use of measurement instruments. The tailor-made education should in particular focus on knowledge of measurement instruments, integration in daily routine, and guidelines on when to use *which measurement instrument *for *which patient *and information on the interpretation of the scores. Furthermore, the interviewed therapists indicated a need for a toolkit of short and feasible measurement tools and, additionally, a short description of how to apply and interpret these instruments.

### Online survey

Completed questionnaires were received from 468 physical therapists; a response rate of 16%. Three hundred ninety four (394) out of these 468 (84%) were private practice physical therapists; 74 (16%) were nursing home physical therapists. The mean age of respondents was 41.6 years (SD = 10). The details of the determinants of respondents are reflected in Table 2. Compared to the private practice therapist, more female and younger nursing home physical therapists responded.

**Table 2:** Determinants of responding physical therapists (online questionnaire)

	Private practices physical therapists	Nurse home physical therapists	National KNGF-data 2005
**Respondents**	n = 394	n = 74	n = 13.355

**Sex (male)**	49.2%	28.6%	49,4%

**Age **(average., SD)**, yr**	41.8 (10.1)	35.5 (9.7)	43 yr

**Work experience **(average., SD), yr	17.9 (10.2)	11.7 (9.0)	

**Number of working hours/wk **(median)	33 or more	25-32	36,7

**Number of patients/wk **(median)*	16-20	6-10	56

**Number of used measurement instruments**			
1-2:	48%	29%	
3-5:	36%	36%	
6-10:	13%	28%	
11-20:	3%	7%	
≥ 21:	0%	n.a.	

**Number of used measurement instruments in**			
0 of every 5 treated patients:	14%	0%	
1 of every 5:	33%	29%	
2 of every 5:	17%	29%	
3 of every 5:	17%	29%	
4 of every 5:	11%	13%	
5 of every 5:	8%	0%	

yr = years; n = number of respondents; SD = standard deviation

KNGF = Royal Dutch Physiotherapy Society [30]

*A substantial number of responding physical therapists were working part-time.

The most dominant facilitators are that the majority of physical therapists indicated having a positive attitude towards the use of standardised measures and being convinced of the benefits of the use of measurement instruments. However, both groups of therapists indicated having difficulty in changing their daily routine. In private practice, 72% of respondents indicated using standardised measures, and 97% of physical therapists working in nursing home indicated using measures. Eighteen different measures could be listed from the group of physical therapists working in nursing homes. Only 5% of these measurement instruments was mentioned once. In contrast, 144 different measures could be listed from the private practice physical therapists, of which 58% were mentioned only once (all based on open questions). These results indicated a higher percentage of measurement use in PTs working in nursing homes. Table 2 presents the different measurement instruments that are generally used by both groups of physical therapists. This demonstrates that, in private practice, a small number of measurement instruments is used frequently.

There proved to be a difference in the most frequently used outcome measures. Table 3 reflects the top-5 of the most frequently listed instruments by physical therapists for each of both settings. In private practice, the first two instruments were very short and easily applicable. The Patient Specific Complaint [23] is comparable to the Patient Specific Functional Scale of Stratford et al. [24].

**Table 3:** Top-five of most frequently used outcome measures by private practice and nursing home physical therapists

Top-5 most frequently used outcome measure in private practice	Number/%	Top-5 most frequently used outcome measure in nursing homes	Number/%
1. Visual Analogue Scale	n = 247 (23%)	1. Berg Balance Scale	n = 20 (19%)

2. Goniometer	n = 77 (7%)	2. 6-minute walking test	n = 15 (15%)

3. Quebec Back Pain Disability Scale	n = 60 (6%)	3. Motricity Index	n = 13 (13%)

4. Patient Specific Complaints	n = 59 (6%)	4. POMA* (Tinetti)	n = 12 (12%)

5. 6-minutes walking test	n = 59 (6%)	5. Functional Ambulation Categories	n = 9 (9%)

*Performance Oriented Mobility Assessment

The survey also investigated the most frequently reported facilitators and barriers to the use of measurement instruments, which are summarised in Table 4. The majority of physical therapists had a positive attitude towards the use of standardised outcome measures and is convinced of the advantages of the use of measurement instruments. Therapists also indicated having difficulties with changing their daily routine.

**Table 4:** The most important barriers and facilitators in the use of measurement instruments, based on the online survey

	Private practice physical therapists	Nurse home physical therapists
**Facilitators**		
- Positive attitude to the use of clinimetrics	85%	97%
- Clinimetrics leaves enough scope for personal considerations	82%	96%
- No resistance against clinimetrics	not mentioned	not mentioned
- Convinced of the benefits of the use of measurement instruments	83%	89%
- Use of instruments to evaluate the effect of a treatment	73%	97%
- Use of measurement instruments enhances the negotiating position to insurance companies	72%	not mentioned
- I already used measurement instruments	97%	not mentioned
- Convinced that use of measurement instruments improves quality of treatment	85%	82%

**Barriers**		
- Changes of daily routine is difficult	54%	32%
- Use of clinimetrics requires extra financial compensation	47%	not mentioned
- Use of clinimetrics takes too much time	44%	14%
- No measurement instruments for diagnostics	63%	23%
- The quantity of measurement instruments makes it difficult to choose the right one	50%	not mentioned
- Application of measurement instruments is not implemented in my clinical reasoning	46%	not mentioned
- No support of management in application of clinimetrics	34%	56%

In summarising Table 4, the main barriers are on the level of the physical therapist (lack of knowledge; problems in attitude) and organisation (lack of time; availability; lack of management support).

## Discussion

The aim of this study was to investigate the current use of measurement instruments, and related barriers and facilitators in the use of measurement instruments in clinical physical therapy practice. Comparing the results of this study with other studies in the literature, the same problems prove to exist in different countries regarding the use of measurement instruments [3,12,13,16,20,25-27]. The results from these different studies do not demonstrate conflicts; there is only sometimes a shift in the emphasis. To get a complete and valid overview of relevant barriers and facilitators, different methods of data collection were used. The additional value of this paper is especially this combination of qualitative and quantitative methods.

A way to classify measurement instruments is the International Classification of Function, Disability and Health Problems (ICF) [28]. Using this framework gives a clearer understanding of the kind of measurement instruments used in clinical practice: focusing on impairments in function, on disabilities, on personal factors or on external factors.

Goniometry and VAS are listed as two of the five standardised measures used by private practice physical therapists, yet they are in the ICF body structure/function category. The five most frequently used measures listed by nursing home physical therapists were in the "activity"-category, but none were in the "participation" category. Physical therapists, particularly in orthopaedic practice, have traditionally focused on the measurement of impairments such as pain, range of motion and muscle strength, but have not utilised standardised measures of activity and participation. Based on our findings, the assessment of activity and participation is clearly not routine in private practice. Perhaps private practice physical therapists deal with more orthopaedic problems which warrant more direct focus on impairments (cure) whereas nursing home physical therapists are focused primarily on functional training (care). In general, the results from Table 3 suggest that physical therapists in private practice use a very small number of different measurement instruments. This is disputable given the fact that physical therapists in private practice, in general, see more patients with different indications each day than therapists in nursing homes. A possible explanation could be the fact that therapists in private practice are more experienced, work more hours per week and see more patients each day. On the other hand, more varied indications should demand wider array of measurement instruments.

A very small number of short and feasible measurement instruments was used by physical therapists working in private practices, where, conversely, a large number of instruments were being used by physical therapists working in nursing homes. This is comparable with the results of the study by Haigh et al. [3] who published a survey on the use of outcome measures in rehabilitation within Europe. However, that survey concerned rehabilitation centres and public hospital institutions, not private practice or nursing homes. Moreover, it concerned not only physical therapists, but also nursing staff, occupational therapists and physicians.

More than 70% of the respondents indicated the use of measurement instruments. More than forty percent of private practice responders indicated using one or two outcome measures and one third indicated using three to five outcome measures in clinical practice. This is comparable with the results of a New Zealand study [13] (40% reported the use of back pain-related outcome measures), a Scottish study [27] (44% of physical therapists in physical therapy departments) and an American study [12] (48% of the responding members of the American Physical Therapy Association) reported to use standardised measures.

The response rate of the survey was substantially lower than reported in comparable study designs [3,12,29]. The responders most likely reflect a group of innovators and early adopters. Also the average age of respondents was below the national average; possibly, younger physical therapists are more likely to use outcome measures. This could indicate an overestimation of the use of measurement instruments and a more optimistic result than exists in reality. The actual use of measurement in daily practice is probably be much lower than 70%.

There are indications from the interviews as well as from the online survey that participants positively reported their use of measurement instruments, meaning there was probably an overestimation of the real application of instruments in daily practice.

Another factor of overestimation is the fact that the online survey as well as the personal interviews are related to a physical therapist's reported or perceived behaviour, which may be different from reality. The semi-structured interviews confirmed the assumption that there is a gap between reported use and perceived behaviour on one hand and reality on the other hand. This is the conclusion of several studies showing that reported use is probably an overestimation of reality in clinical practice [12,13].

The majority of physical therapists indicated having a positive attitude to the use of standardised measures and to be convinced of the benefits of the use of measurement instruments. However, both groups of therapists indicated having difficulty changing their daily routine.

The most important barriers could be detected at the level of the physical therapists (lack of knowledge and insufficient integration in daily practice). In both the interviews and the survey, therapists indicated a need for small scale education, feedback on the use of measurement tools and guidance on which measurement tools to choose. This is consistent with the comparable study for American physical therapists [12] as well as with earlier surveys, in which the majority of participants indicated that the most important barriers are lack of familiarity with, lack of training in and lack of access to (outcome) measures. Other important barriers exist at the level of organisation (lack of time and no instruments available in practice). In addition to this, the interviews in our study demonstrated that in the majority of clinical settings there is no policy on standardised measures. Finally, the lack of computers and software including measurement tools appears to be a barrier.

Earlier studies used mainly written inquiries (questionnaires) to identify barriers and facilitators for the use of measurement instruments in physical therapy [3,12,13]. In this study, we used a combination of a literature study, semi-structured interviews and a questionnaire. It is important to emphasise that the findings of this study concerning the interviews and the online survey are based on the perception of physical therapists. The interviews generated additional information about underlying thoughts and arguments of the therapists for the use or non-use of measurement instruments. Earlier studies focused only on specific outcome instruments. In contrast, we investigated the use of all measurement instruments to gain a complete view and because of the important role of clinimetrics in the process of clinical reasoning (as also indicated in the interviews and the online survey). In general, the results of the interviews and the online survey correspond with the results of earlier studies regarding the use of measurement instruments by physical therapists in the Netherlands [8,9]. However, it must be emphasised that the low response rate to the online survey could influence the validity and generalisability of the generated findings.

The results revealed that the application of measurement instruments is only partially implemented in the process of clinical reasoning in daily practice. The same has been reported by a number of authors across different countries. One reason for this might be that physical therapists have difficulty interpreting measurement scores, as indicated in the interviews, and therefore do not integrate these scores into the process of clinical reasoning. Interpreting scores remains a difficult issue as there are often no normative data available. The use of measurement instruments should not be a goal, but a tool that supports the clinical decision making process. It is therefore, essential to incorporate the use of measurement instruments in the structure of clinical reasoning and daily routine instead of using it as a separate trick. The project group adopted the model for systematic implementation by Grol & Wensing [19] and focused in particular on the first two steps of this model: 1) detection of the improvement goal and 2) analysis of target group and setting. The study findings emphasise the facilitators and barriers for the use of standardised measures by physical therapists in private practice and in nursing homes. The next stage of the study will be the development of implementation tools in order to realise steps 3-5 of this model: selection, testing and evaluation of implementation tools. By classifying the barriers in stages of behaviour change it becomes obvious that it is essential to tailor the implementation strategy: the solution will not be the same for everyone. The results of this study will be used to select and develop different strategies to improve the use of measurement instruments in daily practice. The most important of these implementation tools will be the development of core sets of measurement instruments, making these core sets easily available and the development of tailor-made courses for the implementation and use of standardised measures.

One limitation of this study is the fact that the literature study was not a systematic review of the literature. Another limiting point is the relatively small response rate to the electronic survey, which could jeopardise the validity of the reported findings.

## Conclusions

• A very small number of measurement instruments is used by physical therapists working in private practices, where conversely a large number of instruments are being used by physical therapists working in nursing homes.

• The reported use is probably an overestimation of reality in clinical practice.

• The most important barriers could be detected at the level of the physical therapists (lack of knowledge and insufficient integration in daily practice) and on the level of organisation (lack of time and no instruments available in practice).

• In nursing homes, an important barrier is the lack of support from management.

• There is a need for tailor-made education focusing on implementation in clinical reasoning and organisation structure

• There is a need for a toolkit of short and easily applicable instruments and user descriptions

## Appendix 1: Keywords used for the literature search in PubMed, CINAHL and Cochrane database

Outcome measure

Measurement

Instrument

Assessment

Test

Questionnaire

Clinimetrics

Index

Scale

Reliability

Validity

Sensitivity

Responsiveness

Physiotherapy

Physical therapy

Rehabilitation

and combination of these keywords

## Appendix 2: The Barrier and Facilitators Questionnaire

Barriers and Facilitators Questionnaire*^this questionnaire has not been validated in English.^

The purpose of this questionnaire is to get your opinion on the use of measurement instruments in clinical physiotherapy care during evaluation and treatment. By measurement instruments we mean, for instance, a visual analogue scale (VAS), a goniometer, walking tests, but also questionnaires. Some statements refer to "the project". By this, we mean the Royal Dutch Society of Physiotherapy project on measurement in clinical care.

Below follow a number of statements on the use of measurement instruments. Please indicate whether you completely disagree, disagree, disagree nor agree, agree or completely agree.

I have been using measurement instruments before this project

I have sufficient knowledge to use measurement instruments

I have sufficient skills to apply measurement instruments

Changing my routine is difficult for me

In general, I resist using measurement instruments

I have a positive attitude towards the use of measurement instruments

Using measurement instruments gives me enough room to include patient preferences

Using measurement instruments during treatment is too time consuming

Patients value the use of measurement instruments to gain insight into their functioning

Patients find the use of measurement instruments too time consuming

Co-workers (physiotherapists) support the use of measurement instruments

My supervisor supports the use of measurement instruments

Patients support the use of measurement instruments

The use of measurement instruments fits my way of working in the clinic well

I find using measurement instruments a problem because I do not have (physical) space in my practice

I find using measurement instruments a problem because I have had no training in using them

I would like to know more about the use of measurement instruments before I decide to use them

Using measurement instruments requires additional financial compensation

The use of measurement instruments leaves enough room for me to make my own clinical decisions

Are there reasons, other than the above statements that are barriers for you to the use of measurement instruments? (open question)

Are there reasons, other than the above statements that are facilitators for you for the use of measurement instruments? (open question)

I miss the routine of using measurement instruments in daily clinical practice

In evaluating patients I primarily focus on impairments

Our professional body overloads me with too many guidelines and rules

There are so many different questionnaires; I do not know which one to use.

I am convinced of the usefulness of measurement instruments

In my daily clinical practice sufficient measurement instruments are available

The use of measurement instruments is part of the organisational goals of our practice

The kinds of patients I treat are unsuitable for the use of measurement instruments

The use of measurement instruments is always an integral part of my treatment

I am convinced the use of measurement instruments improves the quality of my treatment

Patients want to evaluate treatment results objectively

Referrers want to evaluate treatment results objectively

Using measurement instruments might strengthen negotiations with insurers

I use measurement instruments primarily for diagnostic purposes

I use measurement instruments primarily for prognostic purposes

I use measurement instruments primarily for evaluative purposes

What would your preferred measurement be?

(please indicate in examples underneath, you can choose more than one answer)

• Impairments in body functions and structures, e.g. Range of motion

Pain

Muscle strength

Swelling

Sensibility

Mental functions (depression etc)

.......................................

• Limitations in activities; e.g. Activities of Daily Life

Standing/sitting

Mobility (e.g. Walking)

Arm/hand (lifting, reaching, grasping)

.................................

• Participation restrictions, e.g. Work

Sport

Social activities

.................................

• Personal factors, e.g. Chronicity

Patient's cognition and attributes

Fear of movement

Coping

..........................................

• Environmental factors, e.g. Problems in home environment

Work conflicts

Financial factors

Sick leave

Stress-provoking factors

Work stress

.......................................

For the evaluation and treatment of patients I use about........different instruments:

- 0 - 2 instruments

- 3 - 5 instruments

- 6 - 10 instruments

- 11-20 instruments

- ≥ 21 instruments

I use measurement instruments during evaluation and treatment in:

- 1 of each 5 patients ( 20%)

- 2 of each 5 patients ( 40%)

- 3 of each 5 patients ( 60%)

- 4 of each 5 patients ( 80%)

- 5 of each 5 patients (100%)

Please indicate your top 5 measurement instruments and for each measurement instrument how often you use this instrument? (example: Roland Morris Questionnaire: 2 out of 19 patients)

Instrument number............of number of patients per week

1...........................................................................

2...........................................................................

3...........................................................................

4...........................................................................

5...........................................................................

Finally, some questions about you and your work environment:

Gender: male/female

Current work environment (if working at more than one setting, please indicate where you see the most patients)

- Hospital

- Rehabilitation Centre

- Nursing Home

- Private Practice

- Other,

How many hours a week do you work as a physiotherapist?

- 0 - 8 hours

- 9 - 16 hours

- 17 - 24 hours

- 25 - 32 hours

- More than 33 hours

How many patients a week do you treat on average?

- 1-5 patients

- 6-10 patients

- 11-15 patients

- 16- 20 patients

- 21-25 patients

- > 25 patients

How many years of experience do you have as a physiotherapist?..................years.

## Appendix 3 Topic-list of barriers and/or facilitators used in the semi-structured interviews

Barriers

Level topics

Physiotherapist

Expertise

Lack of routine in using measurement instruments in evaluation and treatment

Lack of knowledge in using measurement instruments

Lack of education in measurement (instruments)

Diagnostic process mainly directed at impairments

Insufficient experience in using measurement instruments

Attitude/personality

Lack of autonomy, Dutch Royal Society of Physiotherapists directs (what to do and how to do it)

Overwhelmed by evidence based practice

Overwhelmed by quantity of guidelines, rules and information

Habit of determining for oneself what best practice is without outside direction

Insufficiently convinced by need to use measurement instruments and whether this leads to better quality care.

Physiotherapist decides in a different manner whether treatment is successful

Insufficiently prepared to change clinical practice

Resistant to change

Lack of confidence in own (measurement) skills

Organisation

Availability of measurement instruments, guidelines and such

Lack of time

Single person private practice, no feedback colleagues

Monodisciplinary work

Use of measurement instruments not included in organisational policy

Finances

Costs of measurement instruments

Lack of time, time is money

No (additional) reimbursement

Colleagues

Insufficient feedback from, consultation with colleagues

Team not very innovative, 'late adopters'

People break their engagement

Key-persons disagree with the use of measurement instruments

Patient

Expectation patient; does not want measurement, just treatment

Patient category unsuitable for the use of measurement instruments

Patient pressurises physiotherapist

Social context

Overall negative attitude, not ready yet to use measurement instruments

Problems with referrers

Facilitators

Level topics

Physiotherapist

Expertise

Routine in using measurement instruments in evaluation and treatment

Sufficient education in measurement (instruments) and ongoing continuing education

Knowledge of measurement instruments

Attitude/personality

'Readiness to change'

Being convinced of the positive contribution of the use of measurement instruments to the quality of (physiotherapy) care

Understanding the advantages of using measurement instruments

Positive attitude towards the use of measurement instruments

Organisation

Working with (multiple) physiotherapy colleagues

Regular feedback/consultation colleagues

Availability measurement instruments (on paper or digitally).

Use of measurement instruments is part of organisational policy

Colleagues

team is innovative, 'early adopters'

Sufficient support from physiotherapy colleagues

Patient

Patient want objective instruments to evaluate the treatment process

Social context

Positive attitude towards using measurement instruments

Referrers want objective findings/evaluation of treatment

## Competing interests

The authors declare that they have no competing interests.

## Authors' contributions

RS participated in part of the semi-structured interviews and wrote the core text of the manuscript. RvP participated in part of the semi-structured interviews, did the literature search and developed the topic list. HW was responsible for the online survey and data analysis and updated the literature search. JC Conceived of the study and participated in the design and coordination of the study. SB Conceived of the study and participated in the design and coordination of the study and corrected and improved the text of the manuscript. All authors read and approved the final manuscript.

## Pre-publication history

The pre-publication history for this paper can be accessed here:

http://www.biomedcentral.com/1471-2474/12/106/prepub
